# Machine learning liver-injuring drug interactions with non-steroidal anti-inflammatory drugs (NSAIDs) from a retrospective electronic health record (EHR) cohort

**DOI:** 10.1371/journal.pcbi.1009053

**Published:** 2021-07-06

**Authors:** Arghya Datta, Noah R. Flynn, Dustyn A. Barnette, Keith F. Woeltje, Grover P. Miller, S. Joshua Swamidass

**Affiliations:** 1 Department of Computer Science and Engineering, Washington University in Saint Louis, Saint Louis, Missouri, United States of America; 2 Department of Pathology and Immunology, Washington University School of Medicine, Saint Louis, Missouri, United States of America; 3 Department of Biochemistry and Molecular Biology, University of Arkansas for Medical Sciences, Little Rock, Arkansas, United States of America; 4 Department of Internal Medicine, Washington University School of Medicine, Saint Louis, Missouri, United States of America; 5 Center for Clinical Excellence at BJC HealthCare, Saint Louis, Missouri, United States of America; University of Chicago, UNITED STATES

## Abstract

Drug-drug interactions account for up to 30% of adverse drug reactions. Increasing prevalence of electronic health records (EHRs) offers a unique opportunity to build machine learning algorithms to identify drug-drug interactions that drive adverse events. In this study, we investigated hospitalizations’ data to study drug interactions with non-steroidal anti-inflammatory drugs (NSAIDS) that result in drug-induced liver injury (DILI). We propose a logistic regression based machine learning algorithm that unearths several known interactions from an EHR dataset of about 400,000 hospitalization. Our proposed modeling framework is successful in detecting 87.5% of the positive controls, which are defined by drugs known to interact with diclofenac causing an increased risk of DILI, and correctly ranks aggregate risk of DILI for eight commonly prescribed NSAIDs. We found that our modeling framework is particularly successful in inferring associations of drug-drug interactions from relatively small EHR datasets. Furthermore, we have identified a novel and potentially hepatotoxic interaction that might occur during concomitant use of meloxicam and esomeprazole, which are commonly prescribed together to allay NSAID-induced gastrointestinal (GI) bleeding. Empirically, we validate our approach against prior methods for signal detection on EHR datasets, in which our proposed approach outperforms all the compared methods across most metrics, such as area under the receiver operating characteristic curve (AUROC) and area under the precision-recall curve (AUPRC).

## Author summary

Drug-drug interactions account for nearly one-third of adverse drug reactions. Concomitant application of multiple drugs, often used to enhance therapeutic effect and selectivity, can lead to adverse drug reactions of high clinical significance. Commonly used approaches to detect adverse drug-drug interactions often rely on adverse event reporting systems and spontaneous reports. These datasets only contain specific cases where severe reactions were identified and reported by clinicians thereby overlooking a vast majority of unexpected adverse interactions that are often under-reported. The increasing prevalence of electronic health records (EHRs) provides a unique opportunity to mine these datasets and identify previous known and potentially unknown adverse drug interactions. Our proposed logistic regression-based approach identified known and unknown drug-drug interactions from relatively small EHR datasets of about 400,000 hospitalizations. Via performance comparison, our method generalizes better to analysis of EHRs when compared to other common methods currently in use by the U.S. Food & Drug Administration. Our analyses, using this proposed approach, identified 87.5% of positive controls, which are drugs that interact with diclofenac causing an increase in risk for drug-induced liver injury, and identified a novel, potentially hepatotoxic interaction between meloxicam and esomeprazole, which are commonly prescribed together to alleviate NSAID-induced gastrointestinal bleeding.

This is a *PLOS Computational Biology* Methods paper.

## Introduction

Synergistic drug combinations, which consist of at least two active pharmaceutical ingredients, form a crucial therapeutic option for the treatment of complex diseases that may manifest multiple conditions, such as cancer and AIDS [1]. The concomitant application of multiple drugs can enhance therapeutic effect and selectivity, delay drug resistance, allow lower dose of each individual drug and combat multiple related targets to address redundancies in disease mechanisms [2–5]. However, just as multiple drugs can interact in a salubrious manner, they can also interact to cause unintended consequences. Combined drug therapies can result in an antagonistic effect that is smaller than the additive effect of each individual drug or, worse, can result in synergistic toxicity [4]. In some cases, these drug-drug or polypharmic interactions can result in an adverse drug reaction of clinical significance.

Understanding the potentially adverse consequences resulting from drug-drug interactions is a significant problem with regards to patient safety and clinical outcomes. These adverse effects are reflected by the additive risk of each drug the patient is exposed to, as well as how each drug may alter the pharmacokinetic and pharmacodynamic properties of the other co-prescribed drugs [6]. Certain patient groups, such as the elderly, may also be more susceptible due to decreased mobility, increased body mass and impaired renal and hepatic functions [7]. Prevalence of multimorbidity, the co-existence of two or more chronic health conditions, can range from 27.2% of patients to 67% [8, 9]. In the absence of multimorbidity, certain individual disorders, e.g., cancer, can still require a cocktail of drugs to be treated effectively [10]. One recent longitudinal study reported that 35.8% of U.S. adults take at least five drugs concomitantly [11]. Heightened cases of polypharmacy, almost doubling from 8.2% of cases in 1999 to 15% of cases in 2012, have exacted an estimated toll of 177.4 billion USD to treat the resultant adverse polypharmic interactions [12].

In clinical trials, adverse events that can be observed and distinctly mapped to a specific combination of drugs occur at a level of frequency that would require an intractably large patient sample size to detect. *In vitro* and *in vivo* experimental approaches are useful for detecting drug-drug interactions [13–16], but at an increased expense in terms of resources, monetary cost, labor and time relative to computational approaches. A set of *N* drugs would require evaluation of *N*(*N* − 1)/2 pairwise drug combinations. As the number of co-administered drugs increases, there is a combinatorial explosion of possible pairwise drug combinations. In contrast, computational approaches are appealing for rapid, high-throughput screening and early detection of adverse drug-drug interactions. Furthermore, computational approaches can incorporate multiple data sources that increase availability to a wider range of population subgroups and to long-term, post-approval therapeutic contexts not explored in short-term clinical trials [17].

Previous research studies have focused on ranking drug–drug event associations using public databases and spontaneous reports [18]. There exist several data mining algorithms that generate and rank adverse drug associations, or signals, based on projections of the data to two-dimensional contingency tables. Such methods include relative risk (RR), proportional reporting ratio (PRR) and reporting odds ratio (ROR) [19, 20]. More complex dis-proportionality methods build on top of the aforementioned statistical measures of association. Namely, Multi-item Gamma Poisson Shrinker (MGPS) is widely used and is the U.S. Food Drug Administration’s main signal detection algorithm for pharmacovigilance [21].

MGPS is conceptually similar to PRR, but incorporates Bayesian shrinkage to produce dis-proportionality scores that alleviate variability issues with limited data and small case numbers [21, 22]. MGPS assumes that the number of observed counts of a drug combination and adverse event pair is drawn from a Poisson distribution with an unknown mean that can be computed as a function of λ. The goal is to estimate the λs. Each λ is assumed to be drawn from a common, 5-parameter prior distribution, which is further assumed to be a mixture of two gamma distributions. Using an empirical Bayes approach, the 5 parameters are estimated such that they maximize the marginal likelihood and empirical Bayesian geometric mean (EGBM) scores are output for each λ [21–23].

Bayesian confidence propagation neural networks (BCPNN) also take a Bayesian approach to signal generation [24]. BCPNNs are similar to feed-forward neural networks, but Bayesian principles are used during learning and inference. Other popular techniques, such as Bayesian logistic regression, have also been used to analyze the effects of drugs in pharmacovigilance studies [25]. Beyond the scope of this study, there also exist methods that operate outside of EHRs and incorporate additional data sources to model polypharmacy at a network level. Recently, Burkhardt *et al.* [26] have proposed the use of neural embeddings to predict adverse drug-drug interactions. Another method, Decagon, achieves strong performance on polypharmacy effects with a strong molecular basis via data sets on protein-protein interactions, drug-protein target interactions and known polypharmacy side effects [27]. Approaches such as DeepDDI [28] have been developed to study drug interactions from structures of chemical compounds but have not used EHR datasets and often lack interpretability.

The primary disadvantage of these approaches is that they rely on incomplete datasets, studying specific cases where severe reactions were identified and reported. Though these datasets are large, they are often biased in ways that limit the interpretability, certainty and robustness of results derived from them. For example, an increase in reports of adverse events associated with a drug could be caused by an increase in prescriptions for that drug.

In this study, we propose a logistic regression-based machine learning algorithm that infers drug-drug associations from EHR datasets. The EHR datasets include several avenues of information that have not yet been fully exploited. It is also not biased towards adverse events, since it includes all hospitalizations with and without adverse events. To clarify, the previously mentioned methods are most widely used within the context of spontaneous reporting systems, which primarily collect reports of adverse events made by clinicians or patients to a regulator or product manufacturer [23]. Furthermore, since our model takes into account outcomes from all hospitalizations, it does not suffer from potential under-reporting of unexpected adverse interactions, which is otherwise a common source of signal loss [29].

We hypothesize that statistical modeling on EHR data can identify drug-drug interactions. Our proposed model simultaneously reveals the risk contribution of individual drug and pairs of interacting drugs with respect to a therapeutic outcome, such as an adverse event. Empirically, we have shown that our model can extract meaningful drug-drug associations between a candidate drug, whose potential drug-drug interactions are of interest, and all of its co-prescribed drugs in EHR datasets consisting of less than 400,000 hospitalization records.

As a case study, we have identified drug-dependent risk of nonsteroidal anti-inflammatory drugs (NSAIDs) with respect to drug-induced liver injury (DILI). NSAIDs are one of the most commonly and widely used class of drugs, yet many of them have been implicated in causing adverse drug reactions [30]. Since NSAIDs are often used, concomitantly, with a variety of co-prescribed drugs across a wide range of therapeutic contexts, the resultant polypharmic interactions may drive some of these adverse drug reactions. Furthermore, NSAIDs are an ideal class of drugs for such a case study, because they are prescribed in a wide variety of contexts and it is anticipated that their widespread use may allow the detection of statistically significant interactions.

## Materials and methods

### Study population and study design

The electronic healthcare records (EHR) dataset contains data of 397,064 hospitalizations reported by the BJC HealthCare system in St. Louis, Missouri, USA (Table 1) [31]. The 397,064 hospitalizations involve 223,883 unique patients. The earliest inpatient admit date was September 2012 and last discharge date was October 2016. The number of hospitalization cases in the St. Louis area during the data collection period determined the sample size. The hospitalization cohort (aged ≥ 18 years) contains 176,443 (44.44%) male hospitalizations, 189,723 (47.78%) female hospitalizations and 30,878 (7.77%) hospitalizations with no specified gender. The cohort’s median age is 63.2 years (max: 110.4; min: 17.9) and the median hospital stay is 3 days (max: 214; min: 0). Each hospitalization is associated with demographics, diagnoses (23366 ICD9, 10 codes), drugs (1083 unique active ingredients) and procedures (13097 ICD 9-CM, 10-PCS codes). In this study, we included drugs that were administered orally or via intravenous route.

**Table 1:** Characteristics of hospitalizations in cohort.

Characteristic	Quartile	Median (Min, Max)	%DILI positives
Age (years)	Q1	82.2 (74.5, 110.4)	8.6 (1038)
	Q2	68.5 (63.2, 74.4)	9.9 (1193)
	Q3	57.7 (51, 63.2)	9.7 (1169)
	Q4	39.2 (17.9, 50.9)	9.9 (1192)
Length of stay (days)	Q1	8 (5, 214)	48.8 (5866)
	Q2	4 (3, 5)	24.7 (2966)
	Q3	2 (2, 3)	15.5 (1857)
	Q4	1 (0, 2)	11 (1324)
No. of drugs	Q1	22 (17, 101)	42.4 (5092)
	Q2	15 (13, 17)	23.5 (2824)
	Q3	11 (9, 13)	19.1 (2291)
	Q4	6 (1, 9)	14.6 (1750)
No. of diagnoses	Q1	24 (19, 88)	48.8 (5861)
	Q2	16 (13, 19)	26.3 (3157)
	Q3	11 (8, 13)	17.2 (2063)
	Q4	6 (1, 8)	7.8 (933)

% DILI positives are based on the total DILI positives in the data set. % DILI positives may not sum to 100% due to missing values.

As a case study of our proposed modeling framework, our study design compared hospitalization records involving the presence or absence of DILI and evaluated the model’s ability to, using these comparisons, derive drug dependent DILI risk that corresponds with knowledge from literature or public databases. To train our proposed modeling framework, each datapoint was a hospitalization with specific admit and discharge dates. Hence, it is quite plausible that one patient with multiple hospitalizations over time will contribute multiple datapoints to the training set. In order to capture drug interactions during a specific timeline, we performed hospitalization-based analyses rather than a patient-based analyses. A major drawback with patient-based analyses is that there can be significant time differences between two successive hospitalizations and drugs administered during the first hospitalization will, in no plausible way, interact with drugs administered during the second hospitalization. A hospitalization-based analyses addresses this issue, since we can now capture meaningful drug interactions within a specific hospitalization and not across different hospitalization timelines.

### Polypharmacy data: Twosides database

We downloaded the v0.1 release of the Twosides database, which contained data on drug-drug interaction side effects reported up to, and including, the year 2014 [32]. Twosides is based on analysis of drug-drug interactions mined from the FDA Adverse Event Reporting System (FAERS). In this study, we primarily utilized Twosides to understand the validity of the model’s predictions in the context of known polypharmic toxicity. During analysis of a specific NSAID, we extracted only those Twosides interactions that involved the NSAID with conditions related to hepatotoxicity: DILI, liver injury, hepatocellular injury, mixed liver injury and cholestatic liver injury. To extract positive and negative controls for comparison with our model’s results, we used the proportional reporting ratio (PRR) recorded for each Twosides interaction. The PRR is used as a signal of the drug pairs side-effect association. A PRR of 2 suggests that the adverse event is reported twice as frequently as for individuals receiving co-administration of the drug pair relative to taking the drug alone. For positive controls, we only considered interactions with a PRR equal to or greater than 5. For negative controls, we only considered interactions with a PRR less than 1.

### DILI definition

The DILI outcome was computed using a combination of diagnoses and procedure codes, available for each hospitalization. The codes are defined in accordance with the International Classification of Diseases (ICD), which has near-universal availability in EHR systems [33]. DILI can be present with a wide range of severity, from mild and reversible elevation of liver enzymes to permanent liver failure. Mild DILI is more common, not usually reported to the FDA, but well represented in the study population and has a large impact on healthcare costs by increasing length of stay at the hospital. Moreover, most DILI cases result from dose-independent, idiosyncratic injury [34, 35] and similar underlying mechanisms may be present in both mild and severe DILI. As an example, metabolite reactivity commonly causes rash, but can also cause rare, severe hepatotoxicity by the same bioactivation mechanism [36]. Thus, adverse reactions which cause mild DILI may also be associated with severe DILI. For these reasons, we used a definition of DILI that also included low severity cases.

Hospitalizations were deemed “DILI positive” under fulfillment of the following three criteria: (A) having diagnosis codes that indicate the presence of DILI, such as (1) elevation of levels of transaminase, lactic acid dehydrogenase and serum enzymes, (2) poisoning by aromatic or non-opioid analgesics, antipyretics and antirheumatics causing adverse effects in therapeutic use, (3) toxic liver diseases such as cholestasis, hepatitis and hepatic necrosis; (B) not having diagnosis codes that include (1) poisoning by, adverse effect of and under-dosing of systemic antibiotics, (2) alcoholic liver diseases, internal injury to liver and inflammatory liver diseases, (3) malignant neoplasm of gallbladder, hepatic bile ducts and small intestine, (4) pancreatic diseases; (C) not having procedure codes involving (1) surgeries on liver such as marsupialization of liver lesion, hepatectomy, lobectomy, laceration, etc., (2) surgeries on gallbladder and biliary tract including cholecystotomy, cholecystostomy, anastomosis, etc. and (3) surgeries on pancreas such as pancreatotomy, marsupialization of pancreatic cyst, transplantation of pancreas, etc. Applying the aforementioned definition, we identified 12,014 hospitalizations associated with DILI.

### Estimating percent relative effect

In this study, we have reported the effects of drug-drug interactions on DILI outcomes in terms of percent relative effect. We used odds ratio from our models to approximate the relative risk of the independent and candidate drug dependent interactions. In epidemiology, relative risk, or the risk ratio, is defined as the ratio of probabilities of an event in the exposed group to that in the non-exposed group. Odds ratio (OR) is defined as the ratio of the odds of an event in the exposed group to the odds of that event in the non-exposed group. In our dataset, the number of DILI negatives greatly outweighs the number of DILI positives. Hence, we estimated the relative risk as

$$\begin{array}{c} RR=\frac{\text{probability}\;\text{of}\;\text{DILI}\;\text{in}\;\text{exposed}\;\text{group}}{\text{probability}\;\text{of}\;\text{DILI}\;\text{in}\;\text{non-exposed}\;\text{group}}=\frac{\left(\frac{a}{a+b}\right)}{\left(\frac{c}{c+d}\right)}\approx \frac{\left(\frac{a}{b}\right)}{\left(\frac{c}{d}\right)}=OR \end{array}$$ (1)

where *a* and *b* are the respective number of events (DILI positives) and non-events (DILI negatives) in the exposed group and *c* and *d* are the respective number of events and non-events in the non-exposed group. A risk ratio greater than one suggests an increased risk of DILI in the exposed group, whereas a risk ratio less than one suggests a reduced risk of DILI in the exposed group. Finally, we have computed the percent relative effect (the percent change in the exposed group). In essence, we have considered the non-exposed group as having 100% of the risk and express the exposed group relative to that.

$$\begin{array}{c} \%\;\text{increase}\;\text{or}\;\%\;\text{decrease}\;\text{in}\;\text{relative}\;\text{effect}=\pm (RR-1)\times 100 \end{array}$$ (2)

where the (+) sign indicates an increase in percent relative effect and a (−) sign indicates a decrease in percent relative effect in the exposed group.

### Drug interaction network (DIN)

We have used a logistic regression model to estimate the independent and dependent risk of drugs relative to an outcome variable. Rather than estimating the full pairwise matrix of interactions, the model learns the risk dependent on a single candidate drug, whose potential interactions with other drugs are of interest. Equivalent to learning a single column of a pairwise interaction matrix, this approach dramatically reduces the number of weights to be learned, focusing all modeling effort on a more focused question—what is the independent risk of each drug and what is the additional risk when co-prescribed with the candidate drug?

**Fig 1:**
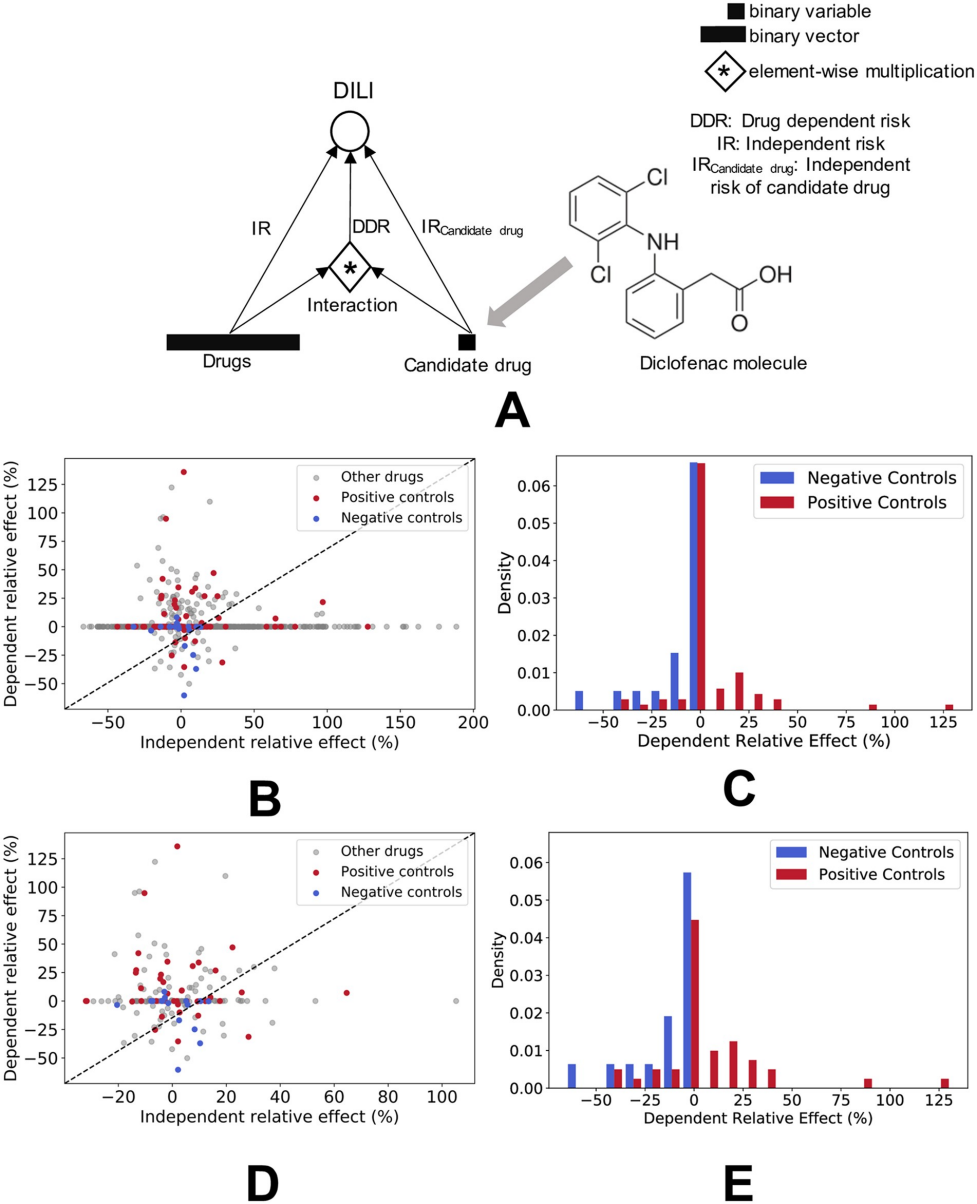
Illustration of model architecture and framework for assessing independent and dependent relative effects of drugs. (A) Model architecture for our proposed modeling framework using logistic regression. (B) Variations between independent and dependent relative effect of drugs. Red and blue respectively correspond to positive and negative controls used during the evaluation of diclofenac dependent risk and DILI. Grey corresponds to all other drugs in the hospitalization cohort that were co-prescribed with diclofenac. (C) Distribution of the Twosides-derived positive and negative controls, with respect to model output for diclofenac. The peak around 0 is suspected to be due to a lack of co-occurrence data for those drugs. (D) Variations between independent and dependent relative effect for diclofenac, after elimination of drugs that did not surpass a diclofenac co-occurrence threshold of 10. (E) Distribution of the Twosides-derived positive and negative controls, after elimination of drugs that did not surpass a diclofenac co-occurrence threshold of 10.

The logistic regression model has two branches: an independent risk branch and a dependent risk branch (Fig 1A). The input to the independent risk branch is a binary vector that records whether or not a drug was administered during the hospitalization. The input to the dependent risk branch is the same vector when the candidate drug is prescribed in the hospitalization, otherwise it is a vector of zeros. Conceptually, the presence or absence of the candidate drug acts as a switch that controls the input to the dependent risk branch. Mathematically, the input to the dependent risk branch is computed as an element-wise multiplication between the binary vector representation of a hospitalization and a binary scalar variable denoting the presence (binary scalar variable is 1) or absence (binary scalar variable is 0) of the candidate drug in that hospitalization. The logistic regression model uses the inputs from both of these branches to estimate the probability of the outcome variable, e.g. DILI in this study, using the maximum likelihood estimation framework. The coefficients, learnt by the model, are then used to compute the percent relative effects of drugs when prescribed independently and co-prescribed alongside the candidate drug of interest, respectively.

Though not considered in this study, we expected that improvements are possible. We point out that continuous variables, such as age, were not used as an input feature in our modeling framework and we only used the binary encoding of presence (represented by 1) or absence (represented by 0) of drugs during a hospitalization timeline as input to our models. For example, encoding the severity of DILI as distinct outcomes would give the model additional information that may yield better estimates. Likewise, encoding the dose for each drug would also reduce noise. We also expected that using a dependent risk input vector for drugs, that are administered on the same days during a hospitalization, would produce better estimates, as drugs without overlapping exposures do not usually interact. However, it appears that these improvements were not necessary to produce clinically relevant results.

## Results & discussion

We have evaluated the proposed framework’s capabilities on three tasks as a demonstration of its utility. We studied the role of diclofenac in hepatotoxicity across the full range of drugs co-prescribed with it in our clinical dataset. We also demonstrated that the model can elucidate a specific hypothesis concerning meloxicam and CYP 3A4 inhibitors. Finally, we ranked the overall hepatotoxic risk of eight commonly prescribed NSAIDs. Where applicable, we also compared the model against several common methods for EHR signal detection.

### Diclofenac dependent risk and DILI

The risk of liver injury with NSAIDs is normally not substantive. Clinical incidence of severe liver injury, resulting from NSAIDs, is 1–10 cases per 100,000 prescriptions [37], with NSAIDs being widely used and clinically ubiquitous. Less severe DILI with mildly elevated liver enzymes is much more common. Moreover, association of NSAIDs with other hepatotoxic drugs is marked with elevated hepatotoxic risk [38, 39]. Potentially, hepatotoxic medications taken simultaneously with NSAIDs may result in a six to nine times increase in frequency of liver injury [40]. In particular, diclofenac is the most common NSAID associated with hepatotoxicity. In fact, 34.1% of hepatotoxic cases associated with NSAIDs involved the use of diclofenac [41].

To analyze diclofenac’s involvement in DILI risk, we trained a model to estimate both independent risk (IR) and diclofenac dependent risk (DDR) of a given drug. The model finds an association between the coefficients of the inputs and how informative each input vector and co-prescribed drug is in predicting the DILI risk target—the higher the coefficient, the higher is the association. The model’s 10-fold cross-validation AUC is 0.68 ± 0.009, with a low standard deviation indicating that the model is not overfit. After the training phase, we evaluated the model on the hospitalization cohort and computed the IR and DDR for the remaining unique active ingredients. Fig 1B visualizes the distribution of IR and DDR associations learned by the model for all drugs present in the hospitalization cohort.

Diclofenac is known to independently cause hepatotoxicity. Hence, most drugs co-administered with diclofenac, in cases that result in DILI, are themselves not likely to be the culprits in causing a DILI outcome via interactions with diclofenac. As expected, Fig 1B shows that the majority of the drugs do not have a positive DDR with respect to DILI risk, regardless of their IR. Nevertheless, two drugs that independently cause hepatotoxicity could combine synergistically to have a stronger hepatotoxic effect. The model identifies a few such drugs that have both a positive IR and a positive DDR that is greater than the drug’s IR. Unsurprisingly, there are also few interactions that have a positive IR and negative DDR, which signifies that, individually, hepatotoxic drugs do not become safer in the presence of diclofenac. Going forward, the drugs of most interest will be those that possess low IR but high DDR.

To evaluate the model, we used diclofenac interactions from Twosides as a reference to extract 71 positive controls and 20 negative controls that are also reported in our EHR data. The distribution of model scores, binned by control type, is shown in Fig 1C. On initial inspection, the model not only indicates potential high-priority diclofenac interactions, but also a relatively high density of drugs with DDR as zero. Since output of DDR as zero may be influenced by a lack of co-occurrence between diclofenac and a given drug, we also filtered out drugs below a co-occurrence threshold and replot the scatterplot and histogram in Fig 1D and 1E, respectively. Based on rationale from prior literature, we set the co-occurrence threshold to 10 [42]. As expected, filtering drugs by a co-occurrence threshold lowers the peak. It is to be noted that the peak for positive controls is lowered more than the peak for negative controls. Thus, there is a greater proportion of positive controls than negative controls that are assigned to DDR values as zero, based on an absence of co-occurrence in the data. Likely, the negative controls are not assigned DDR of 0 because of a lack of co-occurrence but because the reported co-occurrence often results in a negative DILI outcome.

To understand how well the model’s top predictions align with Twosides, we focussed on the top 20 diclofenac interactions from Twosides, sorted by PRR. Of the 20 co-prescribed drugs, 4 were not present in our EHR data. Of the remaining 16 co-prescribed drugs, 14 of the interactions had a positive dependent relative effect (Table 2). The remaining 2 interactions might have been missed due to a limitation in data availability. In our EHR data, bisoprolol and rivaroxaban each had 0 hospitalizations that involved a DILI positive case with diclofenac co-prescription. In contrast, the Twosides data set contains 3 DILI positive hospitalizations that involved co-administration of rivaroxaban and diclofenac and 6 DILI positive hospitalizations that involved co-administration of bisoprolol and diclofenac.

**Table 2:** Assessment of positive and negative controls for diclofenac model validation.

Co-prescribed Drugs	Dependent Relative Effect (%)	Twosides PRR	O+ Rx+	O- Rx+	O+ Rx-	O- Rx-	Control
Lidocaine	47.1	30	4	27	1311	17705	+
Amoxicillin	35.0	40	1	0	103	1884	+
Acetaminophen	34.6	40	12	240	3260	93351	+
Olmesartan	33.8	20	2	16	79	2574	+
Aspirin	27.1	20	11	295	3840	136147	+
Omeprazole	26.8	0.91	3	53	325	11248	-
Pioglitazone	26.8	20	1	0	23	739	+
Famotidine	16.6	20	6	149	2248	73609	+
Carbamazepine	11.2	16.7	1	9	56	2205	+
Cefazolin	9.14	30	4	95	1688	53881	+
Simvastatin	8.13	0.63	5	130	917	31079	-
Esomeprazole	7.14	23.3	5	70	3023	51870	+
Escitalopram	6.51	20	2	39	369	11764	+
Tamsulosin	0.12	30	1	27	135	7805	+
Enoxaparin	0.017	22.5	11	304	2667	87483	+
Oxazepam	0.07	30	0	0	0	18	+
Folic Acid	-0.003	0.67	1	37	1249	33131	-
Rivaroxaban	-0.007	20	0	10	53	2976	+
Celecoxib	-0.03	0.91	0	12	177	9248	-
Adalimumab	-0.13	0.91	0	0	0	2	-
Bisoprolol	-0.32	20	0	6	17	573	+
Amlodipine	-3.19	0.63	2	67	1293	35437	-
Dexamethasone	-24.9	0.83	0	24	1053	19860	-
Morphine	-37.1	0.71	6	212	3241	84935	-

The top 20 interactions, by PRR, were extracted from our filtered Twosides data set and are used as positive controls. Of the 16 positive controls, 14 were successfully captured by the model. The 2 uncaught positive controls reflect a limit in the data availability, as neither positive control had any cases of DILI and diclofenac co-administration from which the model could learn an association. We also extracted 8 interactions with PRR <1 to be use as negative controls. Of the 8 negative controls, 6 where successfully captured by the model. O+ and O- designates the DILI outcome’s presence and absence, respectively. Rx+ and Rx- designates whether diclofenac is prescribed or not. Grayed out rows indicate diclofenac-drug interactions that may be undersampled based on a co-occurrence threshold of 10.

In addition, we extracted the bottom 10 diclofenac interactions from Twosides; 8 of which were present in our EHR data. 6 of the 8 interactions had a negative dependent relative effect. One explanation for the 2 missed negative controls is that, depending on the available data in our EHR datasets, it is possible for the model to learn differing associations between drug-drug interactions, compared to those associations that can be extracted from Twosides. Whereas the EHR data set and Twosides each report single-digit DILI positive hospitalizations involving diclofenac with simvastatin and omeprazole, Twosides DILI negative hospitalizations are a magnitude greater compared to the EHR data set’s DILI negative hospitalizations (10^3^ for Twosides compared to 10^2^ for our EHR data set). Regardless, the model’s results are statistically significant for both positive and negative controls via a two-sided Fishers exact test (*p*-value < 0.01).

Afterwards, we also examined the top 20 DILI interactions predicted by the model, as sorted by the dependent relative effect. Of these 20 interactions, 12 had a clinical basis reported in the Twosides dataset (Table 3). The other 8 prescribed drugs were not in the Twosides dataset for the interactions we used to filter DILI outcomes.

**Table 3:** The top 12 diclofenac interactions, as predicted by the model.

Co-prescribed Drugs	Percent Dependent Relative Effect	Twosides PRR	O+ Rx+	O- Rx+	O+ Rx-	O- Rx-
Ciprofloxacin	136	10	6	43	921	22878
Fluoxetine	96.3	3.33	3	36	258	9924
Cetirizine	95.0	2.5	4	58	351	12576
Atorvastatin	94.8	10	5	75	1390	48234
Ondansetron	50.6	5	11	236	2550	76289
**Meloxicam**	**48.3**	**3.33**	2	18	93	4457
Lidocaine	47.1	30	4	27	1311	17705
Metformin	42.0	10	3	62	253	11260
Topiramate	41.2	2	2	25	44	2423
Amoxicillin	35.0	40	1	0	103	1884
Acetaminophen	34.6	40	12	240	3260	93351
**Olmesartan**	**33.8**	**20**	2	16	79	2574

O+ and O- designates the DILI outcome’s presence and absence, respectively. Rx+ and Rx- designates whether diclofenac is prescribed or not. Grayed out rows indicate diclofenac-drug interactions that may be undersampled based on a co-occurrence threshold of 10.

We further cross-referenced each interaction with results from the literature. Several of the co-prescribed drugs in Tables 2 and 3 have varying degrees of known hepatotoxic associations. The co-prescribed drugs with reported DILI association in literature include acetaminophen [43, 44], amoxicillin [45–48], aspirin [49], atorvastatin [46, 50], carbamazepine [45], cefazolin [51], cetirizine [52], ciprofloxacin [50, 53, 54], famotidine [55], fluoxetine [56], metformin [57–59], pioglitazone [60, 61] and topiramate [62, 63]. Not all the reported DILI associations included concomitant consumption of diclofenac, rather combined use of multiple hepatotoxic drugs, such as diclofenac and the aforementioned drugs, is likely to drive Twosides’ reporting of DILI. As an example, the independent relative effect of amoxicillin is 18%, but it becomes more potent in presence of diclofenac and produces a diclofenac dependent relative effect of 35%. Thus, the model can reflect risk for co-prescribed drugs both in presence or absence of the candidate drug.

It is also possible that, in the predicted interactions of positive dependent relative effect, the co-prescribed drug does not promote increased DILI risk. Generally, the co-prescribed drug may not drive the recorded hepatotoxic outcome, but instead can be used during treatments that involve either NSAID administration or the alleviation of hepatotoxic conditions. As an example of the former, co-administration of a proton pump inhibitor, such as esomeprazole, can help to prevent NSAID-associated lesions and damage of the upper gastrointestinal tract [64, 65]. With regards to the latter, lidocaine (Table 2) is a local anesthetic used widely for minor surgeries or invasive procedures. In the absence of supporting literature, lidocaine’s predicted association with diclofenac may instead be due to a polypharmic approach to pain treatment.

Of most interest are those co-prescribed drugs with less independent hepatotoxic association reported in the literature, but with a high dependent risk predicted by the model—such as olmesartan and meloxicam. The model assigns olmesartan, an antihypertensive, with a high dependent relative effect of 33.8% and Twosides also records olmesartan with a high PRR of 20. As a result, future cohort studies regarding DILI may find it valuable to examine the potentially hepatotoxic contexts of olmesartan.

Meloxicam, an NSAID, only has a PRR of 3.33, yet the model predicted a high dependent relative effect of 48.3% for the interaction. Based on reports in the literature, multi-NSAID therapies may provoke increased risk of hepatic injury, in addition to GI bleeding and acute renal failure [66]. It is also possible that, once patients show DILI from diclofenac, they are switched to meloxicam and this change in prescription causes a spurious association. We expect that an improved model, which ensures drugs are co-prescribed at the same time and not just present in the same hospitalization, would resolve this question.

#### Comparison to data mining algorithms: Diclofenac dependent DILI risk

We compared the drug interaction network against several data mining algorithms for signal detection—relative risk (RR), reporting odds ratio (ROR), multi-item Gamma Poisson shrinker (MGPS), and a one-layer Bayesian confidence propagation neural network (BCPNN). We used the EBGM and the 2.5% quantile of the posterior distribution of the information component as statistics to rank signals for MGPS and BCPNN, respectively. For MGPS, we use DuMouchel’s priors as a default [22]. First, we evaluated the drug interaction network (DIN), along with the RR, ROR, MGPS and BCPNN methods, on the 71 positive controls and 20 negative controls used in the case study on diclofenac dependent DILI risk. As an interaction-less baseline, we also assess performance of a logistic regressor (LR) whose input feature vector contains diclofenac and all coprescribed drugs. For this comparison, we computed the area under the receiver-operating characteristic curve (ROC AUC), the area under the precision-recall curve (PR AUC), and the biserial correlation (BC). BC is a variant of point biserial correlation adjusted for an artificially dichotomized variable with some underlying continuity. Table 4 summarizes performance for each method across each metric with 95% two-sided confidence intervals [67, 68].

**Table 4:** Performance metrics comparing drug interaction network to baselines.

Method	ROC AUC	PR AUC	BC
Drug Interaction Network	**80.3% ± 2.5%**	**93.7% ± 1.2%**	0.63 ± 0.050
Relative Risk	57.9% ± 3.7%	83.0% ± 2.3%	0.081 ± 0.033
Reporting Odds Ratio	58.0% ± 3.7%	83.5% ± 2.3%	0.12 ± 0.033
Multi-item Gamma Poisson Shrinker	78.3% ± 2.7%	90.5% ± 1.6%	**0.67 ± 0.046**
Bayesian Confidence Propagation Neural Network	65.9% ± 3.4%	80.9% ± 2.5%	0.49 ± 0.046
Interaction-less Logistic Regressor	60.9% ± 3.6%	87.5% ± 1.9%	0.24 ± 0.033

**Fig 2:**
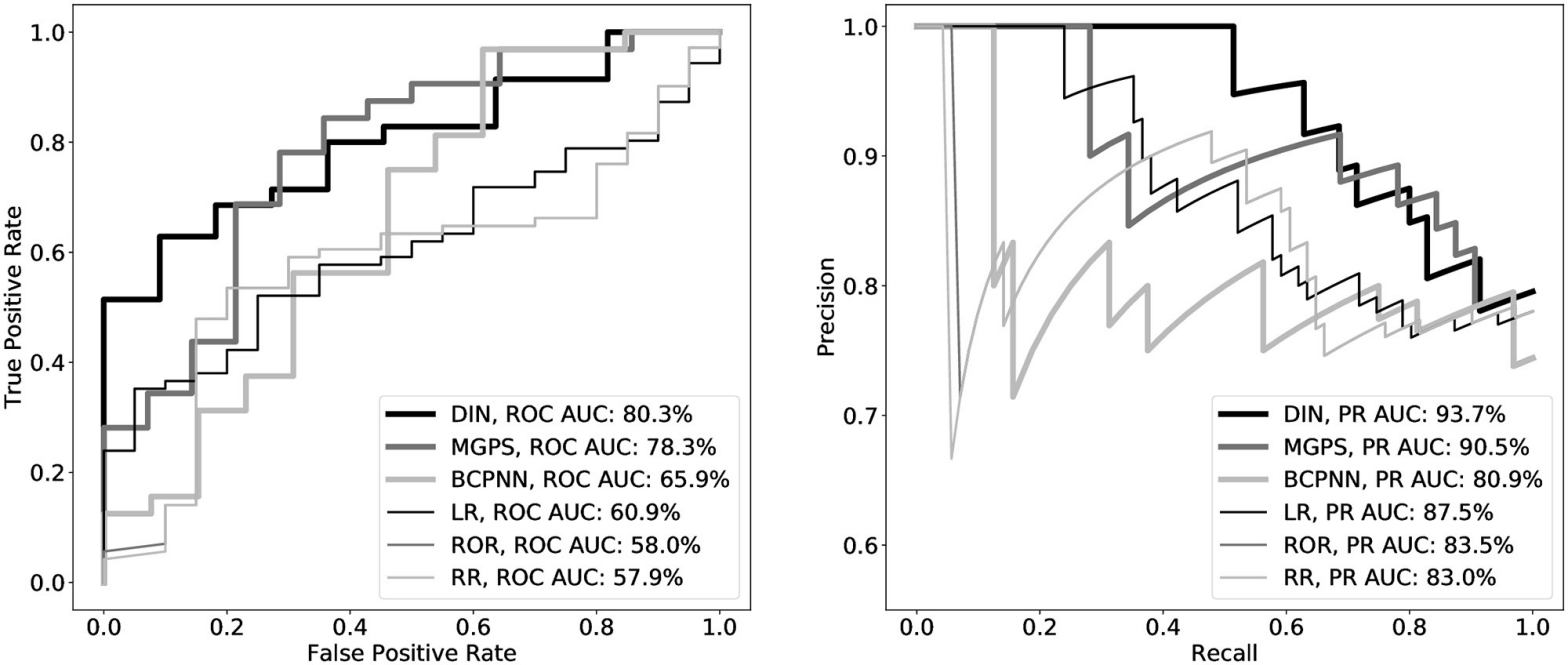
The drug interaction network (DIN) outperforms the compared methods on ROC AUC (left) and, along with MGPS, outperforms the remaining compared methods with regards to PR AUC (right).

The drug interaction network, with a ROC AUC of 80.3% and a PR AUC of 93.7%, outperformed all methods in the comparison (Fig 2). In decreasing order, MGPS, BCPNN, LR, ROR and RR each had a ROC AUC of 78.3%, 65.9%, 60.9%, 58.0% and 57.9%, respectively, and a PR AUC of 90.5%, 80.9%, 87.5%, 83.5% and 83.0%, respectively. Consistent with the ROC AUC and PR AUC performance, MGPS and the drug interaction network also outperformed the remaining methods with respective BCs of 0.67 and 0.63. Though the drug interaction and MGPS were equivalent in terms of ROC AUC and BC, the drug interaction network had a significantly higher PR AUC than MGPS.

Compared to the other methods, the drug interaction network and MGPS did better at extracting relevant signals with respect to adverse events reported in Twosides. This is unsurprising, since both methods are intended to build on top of ROR and RR in a way that mitigates variability issues. BCPNN’s performance on this task should be viewed in light of its intended use cases. The motivation behind BCPNN was to extract drug-adverse event signals on increasing large volumes of spontaneously reported adverse drug reactions [24]. Though BCPNNs may be suitable for handling large data sets, it appears that they are more limited on smaller EHR data sets as analyzed in this case study.

In terms of specific metrics, the drug interaction network and MGPS presented some performance trade offs. The drug interaction network had superior ROC AUC and PR AUC performance compared to MGPS, but MGPS had a better BC. Given routine usage of MGPS as a method of choice for EHR signal detection by organizations such as the US FDA, it is favorable that the drug interaction network outperformed MGPS on ROC AUC and PR AUC and remained competitive on BC [21].

**Fig 3:**
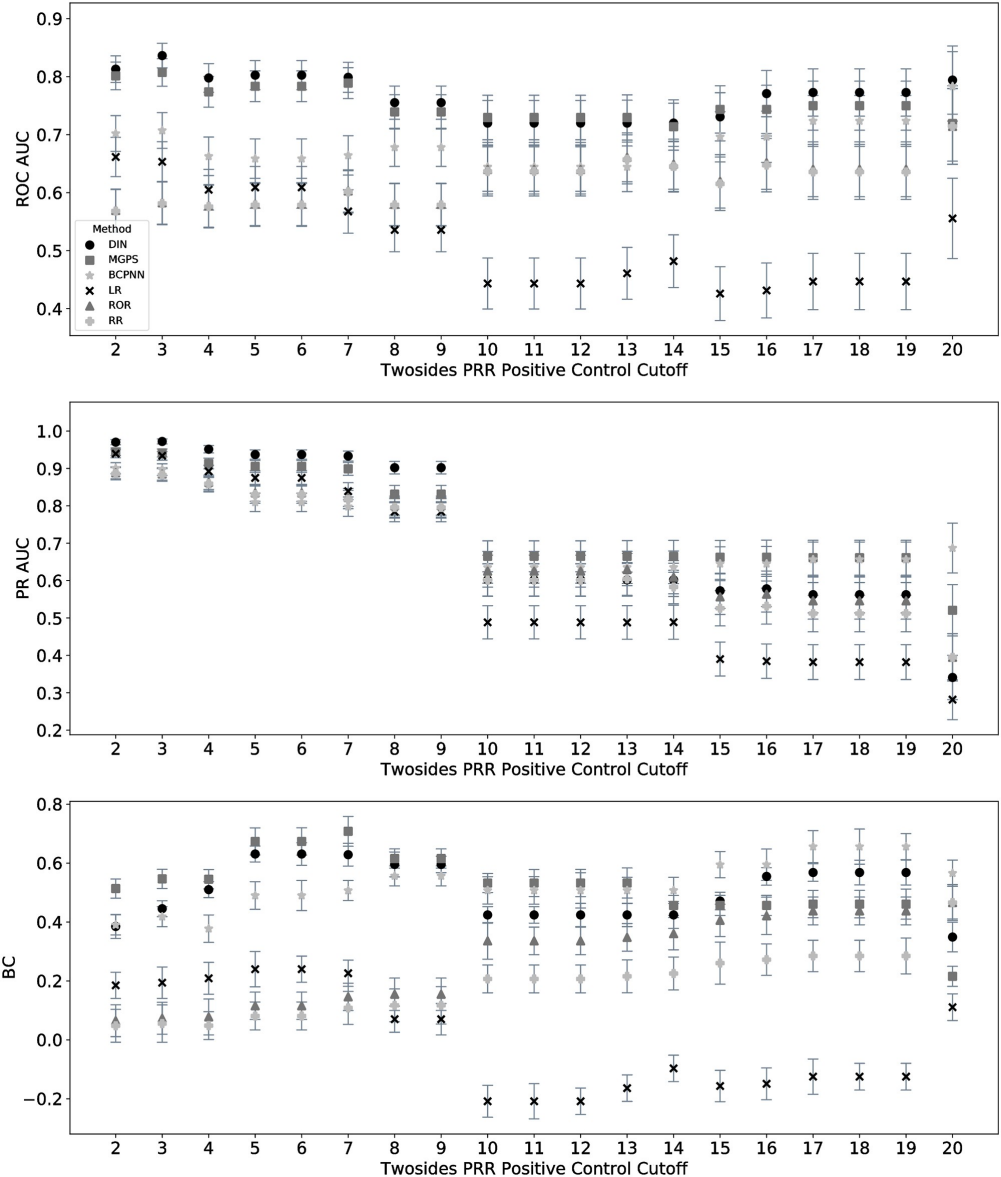
Performance comparison remained consistent up to a PRR cutoff of 10. We examined whether the selection criteria for positive controls has a significant influence on performance by evaluating ROC AUC, PR AUC, and BC for each method over PRR cutoffs of 2 through 20, inclusive. A PRR greater than 10 results in less confident performance estimates and rankings.

A sensitivity analysis identified consistency of the performance comparison trends for different values of the positive control PRR cutoff (Fig 3). The negative control PRR cutoff follows from prior work using Twosides that filtered out DDIs of no interest using PRR cutoffs of 1 [69], but the positive control PRR cutoff of 5 is more arbitrary. We examined whether the selection criteria for positive controls have a significant influence on performance by evaluating ROC AUC, PR AUC, and BC for each method over PRR cutoffs of 2 through 20, inclusive. The error bars represent 95% two-sided confidence intervals [67, 68]. Up to a PRR of 10, results stay consistent with minor deviations but an overall trend of the drug interaction network and MGPS outperforming the other methods. Above a very high PRR of 10, trends become less defined with greater deviations in performance rankings between each method across different cutoffs, narrower separation of the point estimates, and larger confidence intervals.

Though the drug interaction network and MGPS perform competitively on the assessed tasks, the drug interaction network requires less analytical overhead to use for signal detection. Adequate use of MGPS may require estimating priors for the underlying 5-parameter distribution, requiring additional reasoning and work. In addition to priors, decisions must be made for selection of a decision metric, the decision threshold for the decision metric, and the ranking statistic. We achieve strong performance using default settings recommended in literature, but other problem contexts may require further tuning [21, 22]. MGPS also assumes that the number of reports follows a Poisson distribution, which may be at odds with adverse event data sets that can contain many zero count cells. However, this limitation may be temporary as extensions to MGPS continue to be developed [70].

### Metabolic context of a potential and rare hepatotoxic interaction

Previously, we demonstrated the application of the model to diclofenac, one of the NSAIDs most commonly involved in hepatotoxic treatment outcomes. However, other NSAIDs that result in liver injury at much lower frequencies may require a more targeted application of the model. As an example, meloxicam has been associated with hepatocellular damage, but at a frequency of less than 0.1% of severe hepatotoxic NSAID events [71].

Previous studies have shown that meloxicam detoxification pathways are mediated in part by CYPs 2C9 and 3A4 [72, 73]. Therefore, we expected that inhibitors of CYPs 2C9 or 3A4, when co-prescribed with meloxicam, may result in increased incidence of DILI. Consequently, we trained a model to examine meloxicam’s involvement in drug dependent risk with respect to DILI (10-fold CV AUC of 0.68 ± 0.005).

We posit that CYP 3A4 inhibitors may limit meloxicam detoxification. Conversely, CYP 3A4 inducers may expedite meloxicam detoxification. As a result, we first looked at the model’s ability to separate CYP 3A4 inhibitors and inducers based on drug dependent DILI risk. Across 30 CYP 3A4 inhibitors and 17 CYP 3A4 inducers in the data set, the model achieves a ROC AUC of 84.6% and hints at a relation between CYP 3A4 modulators, meloxicam, and DILI risk.

We then inspected the model’s predictions for interactions with co-prescribed drugs that are known CYP 3A4 inhibitors and when used alongside meloxicam, were represented by at least 100 hospitalization records. We cross-referenced the model’s results against known interactions reported by Twosides to see whether the model can garner novel insights (Table 5).

**Table 5:** Predicted interactions between meloxicam and several CYP 3A4 inhibitors.

Co-prescribed Drugs	Percent Dependent Relative Effect	Twosides PRR	O+ Rx+	O- Rx+	O+ Rx-	O- Rx-
Diltiazem	54.8	2.5	9	222	806	21661
**Esomeprazole**	**41.1**	-	10	168	3018	51772
Omeprazole	34.4	2.9	17	493	311	10808
Amiodarone	22.3	10	4	101	921	21396
Ciprofloxacin	8.02	5	6	153	921	22768
Pantoprazole	5.74	1.7	29	1004	3391	97914

O+ and O- designates the DILI outcome’s presence and absence, respectively. Rx+ and Rx- designates whether meloxicam is prescribed or not. Notably, the model predicted a percent relative effect of 41.1% (*p*-value < 0.05) for the interaction involving meloxicam and esomeprazole, which is a known CYP 3A4 inhibitor and not recorded in Twosides. Furthermore, combination use of proton pump inhibitors (esomeprazole) with NSAIDs (meloxicam) to allay potential GI bleeding is common practice [64] and so the clinical relevance of this interaction is high.

Of the 6 CYP 3A4 inhibitors analyzed, 5 of them have some clinical basis in Twosides that links them to DILI outcomes when co-prescribed with meloxicam. The model predicted a percent dependent relative effect of 41.1% (*p*-value < 0.05) for the interaction involving meloxicam and esomeprazole, which is a known CYP 3A4 inhibitor and not recorded in Twosides. Furthermore, combined usage of proton pump inhibitors (esomeprazole) with NSAIDs (meloxicam) to allay potential GI bleeding is a common practice [64] and so the clinical relevance of this interaction is high. Still, validity of this complex interaction would require further clinical investigation. Nevertheless, our model offers a high-throughput, less resource intensive alternative for enumerating hypotheses concerning deleterious drug-drug interactions.

### Comparison of NSAID dependent risk to DILI outcomes

In certain treatment contexts, it is not possible to avoid NSAID use. In general, it would be useful if the model could surmise risk and rank the NSAIDs. Here, we demonstrated how well the model estimates overall DILI percent relative effect for eight NSAIDs. For each NSAID, we trained a separate model to examine that NSAID’s DILI associations. Next, for each NSAID and co-prescribed drug, we constructed a contingency table across two variables: DILI outcome (+ or -) and concomitant NSAID use (+ or -). We only retained significant NSAID and co-prescribed drug interactions, as calculated by Fisher’s exact test. Finally, for each NSAID, we computed the average dependent relative effect (Table 6).

**Table 6:** Ranking the 8 studied NSAIDs by mean percent relative effect.

NSAID	Mean Percent Relative Effect	95% CI	DILIrank Severity Class	Percent NSAID Liver Injury Cases
Indomethacin	56.4%	[32.6%, 80.2%]	8	< 0.1%
Naproxen	48.2%	[23.1%, 73.3%]	3	11.1%
Etodolac	42.9%	[20.7%, 65.1%]	8	< 0.1%
Diclofenac	40.5%	[23.8%, 57.1%]	8	34.1%
Meloxicam	25.3%	[2.18%, 48.5%]	3	< 0.1%
Celecoxib	25.2%	[13.7%, 36.6%]	3	< 0.1%
Ibuprofen	22.4%	[15.8%, 28.9%]	3	14.6%
Ketorolac	21.3%	[14.2%, 28.3%]	3	< 0.1%

Frequencies are based on a prior study derived from 6,023 hospitalizations [71].

The model separates the 8 drugs into two groups based on the mean percent relative effect (*p*-value < 0.1, one-way ANOVA). To validate model rankings, we referenced DILIrank [74] and NSAID-associated DILI outcome frequencies, as reported in the literature [71]. With respect to liver injury cases, diclofenac, ibuprofen and naproxen show high frequencies of 34.1%, 14.6% and 11.1%, respectively. Diclofenac and naproxen belong to the group of NSAIDs with greater predicted DILI association, whereas ibuprofen belongs to the group of lower DILI association. With respect to DILIrank, where a higher severity denotes greater DILI risk, all 3 NSAIDs with high DILI concern and 4 NSAIDs with low DILI concern were correctly grouped. In this case, naproxen stands out as having low DILI concern, yet being grouped with the NSAIDs with greater predicted DILI association.

There is ambiguity on the basis chosen for reference due to each NSAID’s prescription patterns and patient exposure—commonly prescribed NSAIDs will contribute to greater cases of liver injury due to greater exposure. As a result, there is known heterogeneity in studies on liver injury case frequency of NSAIDs [46, 75]. For example, model groupings for indomethacin, etodolac and ibuprofen do not conform to the grouping that results from using the frequency of liver injury cases across NSAIDs. However, of the 8 NSAIDs, ibuprofen is the most commonly prescribed across the EHRs and indomethacin and etodolac are the 2 least prescribed. When grouping the NSAIDs for DILI risk using the DILIrank severity class, model rankings for indomethacin, etodolac and ibuprofen become more clear.

#### Comparison to data mining algorithms: NSAID dependent DILI risk

**Fig 4:**
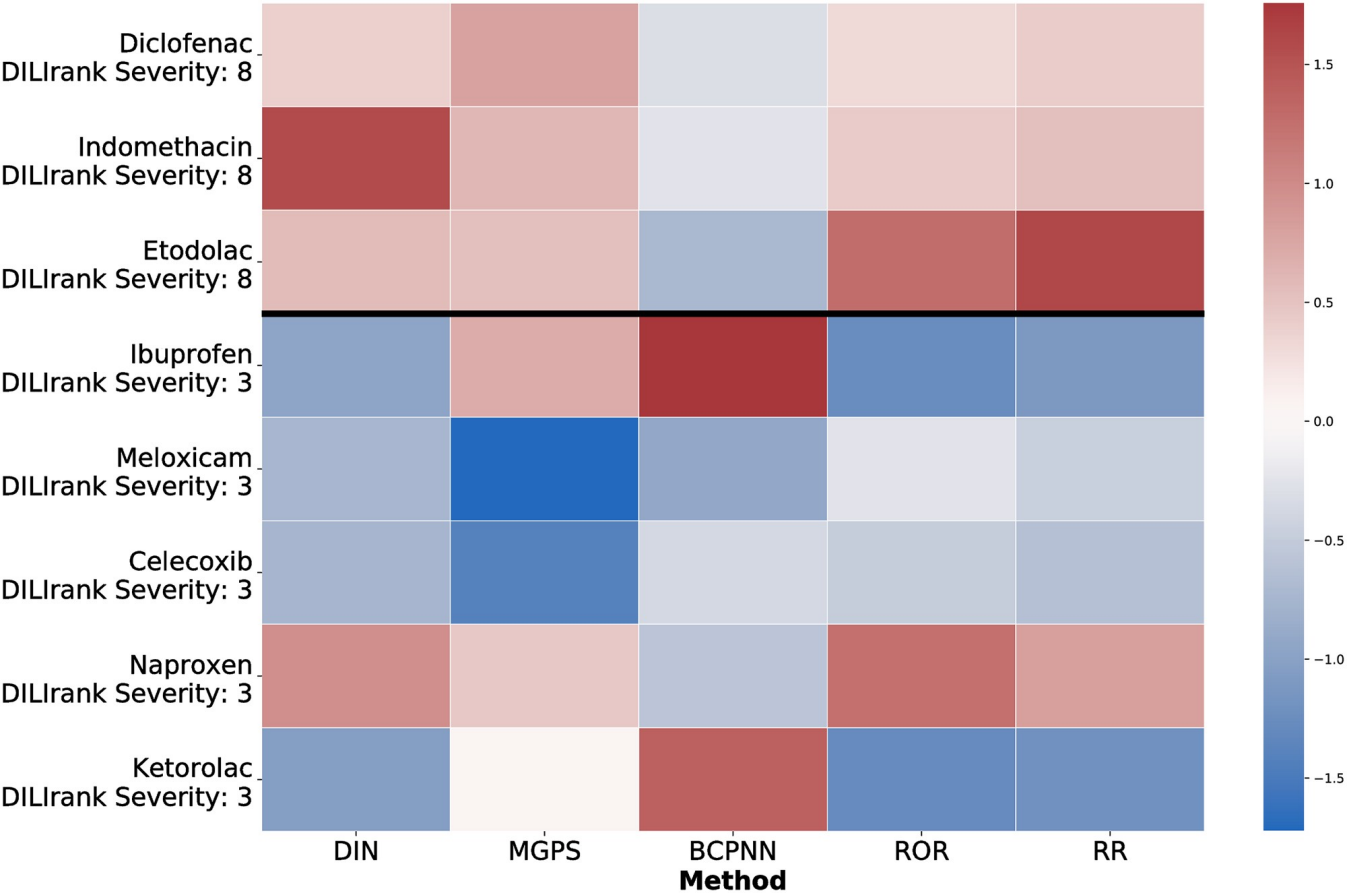
The drug interaction network results in comparable performance with MGPS, RR and ROR on the task of binarizing NSAIDs by DILIrank severity scores. Interestingly, MGPS also assigns high scores to ibuprofen and ketorolac. Though ibuprofen does have DILI risk according to the second binarization reference scheme, ketorolac is indicated as having low DILI risk for both references.

**Fig 5:**
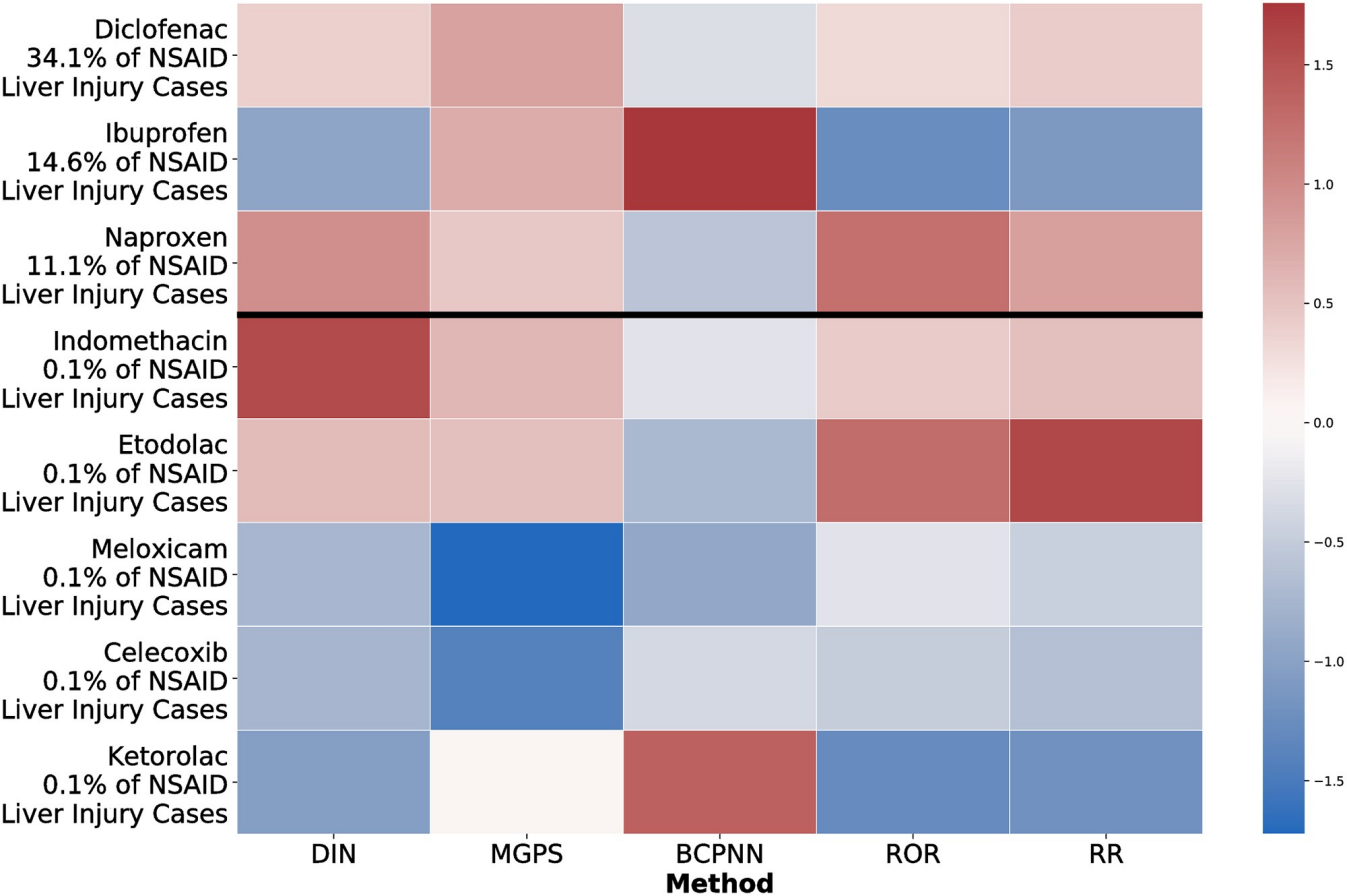
The drug interaction network results in comparable performance with RR and ROR on the task of binarizing NSAIDs by the percentage of NSAID liver injury cases. MGPS is the only method to predict DILI risk for diclofenac, ibuprofen, and naproxen, though, along with BCPNN, it also is the only method to predict DILI risk for ketorolac, which is a false positive for both reference points.

In addition, we also evaluated the drug interaction network and data mining algorithms on the task of ranking the 8 NSAIDs according to DILI risk. For each method, we only retained significant NSAID and co-prescribed drug interactions as calculated by Fisher’s exact test and we output an aggregate NSAID DILI risk by averaging model DILI risk outputs for each NSAID-drug pair. We normalized the aggregate risks for each method and rendered the heat maps in Figs 4 and 5. Each NSAID is binarized into high DILI risk and low DILI risk based on two separate reference points—the DILIrank severity class and the percentage of NSAID liver injury cases reported in a prior study across 6,023 hospitalizations [71].

With respect to the DILIrank severity class binarization, the drug interaction network, RR, ROR and MGPS methods assign high scores to the three NSAIDs with the most DILI risk—indomethacin, etodolac and diclofenac—and to naproxen, which has low DILI risk according to this reference but a high risk according to the percent NSAID liver injury reference. Interestingly, MGPS also assigns high scores to ibuprofen and ketorolac. Though ibuprofen does have DILI risk according to the second binarization reference scheme, ketorolac is indicated as having low DILI risk for both references. Generally, BCPNN does not perform as favorably compared to any of the other methods on this task.

Due to known heterogeneity in studies on liver injury case frequency of NSAIDs [46, 75] and DILIrank’s status as the largest publicly available annotated DILI dataset [74], we place greater weight on the usage of DILIrank as a reference point for NSAID DILI risk. In a comparison of point biserial correlation (PBC) between the model predictions and DILIrank NSAID risk, the drug interaction network and RR outperform the other three methods. The PBC of the drug interaction network, MGPS, ROR, RR and BCPNN are 0.70, 0.54, 0.56, 0.71 and −0.35. The drug interaction network surpasses MGPS, with the biggest distinction between the two being that the latter method assigns high risk to ketorolac regardless of the chosen reference point.

## Model limitations & future directions

One limitation of the current study is due to clinical data availability. For certain drugs, the model yielded positive results, but there was ultimately not enough data available to describe such results as significant. Furthermore, results demonstrated are specific to the patient cohort accessible via the available data. Even if the model’s learned associations don’t always reflect reference datasets or literature, such inconsistencies may instead be a reflection of limited data for certain interactions or of a patient cohort that doesn’t reflect those cohorts used to construct the referential data or literature.

The proposed modeling framework was trained using each hospitalization instance as a datapoint. Hence, one patient, having multiple hospital visits will contribute multiple training instances in the training dataset. This was done to capture meaningful drug interactions within each hospitalization timeline. Concatenating multiple hospitalization timelines into a single datapoint for each patient would lead to interactions between drugs not prescribed in the same time window. However, for rare drug interactions, it may so happen that those are from one patient across multiple hospitalizations thereby leading to poor generalization of results.

In this study, our proposed modeling framework was used as a signal detection algorithm capable of estimating the independent and dependent relative risks of drugs on the clinical outcome. We highlighted the potential utility of our modeling framework in estimating risks of drug exposures from relatively small EHR datasets with known denominators rather than from FAERS database where most incidence rates are estimated with unknown denominators. EHR datasets are an under-utilized resource for studying drug interaction discovery and our research study aims to highlight the benefits of using EHR datasets for this purpose.

The results, presented in this study, have been cross-referenced with other published works as well as previously known interactions from the FAERS database. It is quite plausible that factors such as other comorbidities, other drug exposures both within and outside the hospitalization window and length of hospitalization may confound some findings. A key advantage of EHR datasets for drug interaction discovery is that they contain different data streams such as demographics, hospitalization stay and other drug exposures during a hospitalization timeline whereas adverse reports in FAERS database usually do not contain this additional information. However, in EHR datasets, complex underlying causal relationships exist between different variables and the clinical outcome. Adjusting for these confounding factors was not within the scope of this research study. Future studies include using the drug interaction network in conjunction with the proposed framework by Datta *et al.* [31] to identify and adjust for potential confounding variables. However, for questions in which other pieces of information are necessary, such as drug exposure outside the hospitalization timeline and environmental or behavioral variables, accurate inferences are unlikely to be made solely from EHRs.

Age is often considered an influential confounder in clinical studies involving adverse drug reactions and more than 60% of our hospitalization data did not have any age information associated with them. However, age should not be a confounder for drug interactions which was the key focus of this research study. Also, age was not used as an input variable in our modeling framework in this research study. Furthermore, the findings in this study have been validated using results published in prior studies using FAERS and Twosides databases.

In addition, the manner in which diagnosis, procedure, or other hospitalization codes are used to define possible outcome definitions can lead to ambiguity. Different models can be developed based on the method chosen for applying hospitalization codes or other clinical features, such as the levels of certain aminotransferases or bilirubin, to infer DILI hospitalizations. Ultimately, the method used to define the outcome definition from the available clinical features may depend on the manner in which data was collected for a specific cohort and the target outcome to be studied, e.g., liver, renal, cardiovascular, or other clinical risks.

Lastly, the described approach avoids learning a full pairwise matrix of interactions, which aids in a reduction of learnable parameters and leads to a more focused query. However, multiple models may be required when trying to answer more general queries. Furthermore, a model tasked with predicting many more outputs can lead to a model with better generalization. In future studies, we plan on using interaction detection frameworks [76] for interpreting weights in non-linear extensions to the drug interaction network.

## Conclusion

In this work, we propose a modeling framework to study drug-drug interactions that may lead to adverse outcomes using EHR datasets. As a case study, we used our proposed modeling framework to study pairwise drug interactions involving NSAIDs that lead to DILI. We validated our research findings using previous research studies on FAERS and Twosides databases. Empirically, we showed that our modeling framework is successful at inferring known drug-drug interactions from relatively small EHR datasets(less than 400,000 hospitalizations) and our modeling framework’s performance is robust across a wide variety of empirical studies. Our research study highlights the numerous benefits of using EHR datasets over public datasets such as FAERS database for studying drug interactions. In the analysis for diclofenac, the model identified drug interactions associated with DILI, including each co-prescribed drug’s independent risk when administered in absence of the candidate drug, e.g., diclofenac and dependent risk in the presence of the candidate drug. We have explored how prior knowledge of a drug’s metabolism, such as meloxicam’s detoxification pathways, can inform exploratory analysis of how combinations of drugs can result in increased DILI risk. Strikingly, the model indicates a potentially harmful outcome for the interaction between meloxicam and esomeprazole, confirmed by metabolic and clinical knowledge. Though beyond the scope of this computational study, these preliminary results suggest the applicability of a joint approach—models of drug interactions within EHR data streamlined by knowledge of metabolic factors, such as those that affect P450 activity in conjunction with hepatotoxic events. We have also studied the ability of the model to rank commonly prescribed NSAIDs with respect to DILI risk. NSAIDs undergo widespread usage and are, therapeutically, valuable agents for relief of pain and inflammation. When use of a class of drugs is unavoidable, it is still valuable to select a specific candidate from that class of drugs that is least likely to incur patients’ harm. These results are important because EHR data is increasingly available and may prove to be a more effective approach in mining drug-drug interactions. We believe that the proposed framework in this study will be widely applicable for understanding drug interactions resulting in diverse adverse outcomes using EHR datasets and pave the way for incorporating future analyses based on dosage responses as well as accounting for comorbidities and confounding.

## Supporting information

S1 Data Contains, in separate spreadsheets, the codes and definitions used for each criteria of the DILI outcome definition. (XLSX)

S2 Data Contains, in separate spreadsheets, the underlying numerical data and statistical analysis for Figs 1–5. (XLSX)
